# Medical fitness for work in physicians with psychiatric disorders

**DOI:** 10.1192/j.eurpsy.2024.1712

**Published:** 2024-08-27

**Authors:** S. Chemingui, D. Brahim, I. Youssfi, M. Mersni, M. Methni, H. Ben Said, N. Mechergui, I. Yaich, C. Ben Said, N. Bram, N. Ladhari

**Affiliations:** ^1^Occupational pathology and fitness for work department Charles Nicolle Hospital, tunis, Tunisia; ^2^Occupational pathology and fitness for work department, Charles Nicolle Hospital; ^3^Forensic Psychiatry department, Razi Hospital, La Manouba, Tunis, Tunisia

## Abstract

**Introduction:**

Being a doctor is a profession with special medical requirements. Therefore, the assessment of medical fitness for work among physicians remains a complex decision, particularly for those with psychiatric disorders.

**Objectives:**

To assess the fitness for work decisions among physicians with psychiatric disorders.

**Methods:**

Descriptive and retrospective study including physicians with psychiatric disorders referred to the occupational department of the Charles Nicolle Hospital in Tunis for a medical fitness for work from January 1, 2018 to August 30, 2023.

**Results:**

The study included 28 patients with a female predominance (sex ratio M/F at 0.3) and a mean age of 44.1 ± 12 years. Participants were general practitioners (N=12), junior doctors (N=10), specialists (N=5) and one dentist. They worked in the public health sector in 93% of cases, and had a mean professional seniority of 12.4 ± 9.3 years. A psychiatric history was found in 20 patients. Current psychiatric disorders recorded were: depression (N=15), bipolar disorder (N=7), anxiety-depressive disorder (N=4), personality disorders (N=1) and addiction (N=1). Concerning the fitness for work, six patients were fit for work and 11 were temporarily unfit. Job adjustments were proposed for 11 physicians, mainly night shift exemption.

**Conclusions:**

Physicians are exposed to several occupational hazards and require strict medical qualifications. The impact of psychiatric disorders on medical fitness for work is considerable, and could be avoided by appropriate prevention by occupational health practionnairers, starting from professional orientation.

**Disclosure of Interest:**

None Declared

